# The Advent of Indium Selenide: Synthesis, Electronic Properties, Ambient Stability and Applications

**DOI:** 10.3390/nano7110372

**Published:** 2017-11-05

**Authors:** Danil W. Boukhvalov, Bekir Gürbulak, Songül Duman, Lin Wang, Antonio Politano, Lorenzo S. Caputi, Gennaro Chiarello, Anna Cupolillo

**Affiliations:** 1 Department of Chemistry, Haiyang University, 17 Haengdang-dong, Seongdong-gu, Seoul 133-791, Korea; danil@hanyang.ac.kr; 2 Theoretical Physics and Applied Mathematics Department, Ural Federal University, Mira Street 19, 620002 Ekaterinburg, Russia; 3 Department of Physics, Faculty of Sciences, Atatürk University, 25240 Erzurum, Turkey; gurbulak@atauni.edu.tr; 4 Department of Basic Sciences, Faculty of Sciences, Erzurum Technical University, 25050 Erzurum, Turkey; songul.duman@erzurum.edu.tr; 5 National Laboratory for Infrared Physics, Shanghai Institute of Technical Physics, Chinese Academy of Sciences, Shanghai 200083, China; wanglin@mail.sitp.ac.cn; 6 Synergetic Innovation Center of Quantum Information & Quantum Physics, University of Science and Technology of China, Hefei 230026, China; 7 Graphene Labs, Fondazione Istituto Italiano di Tecnologia, Via Morego 30, 16163 Genoa, Italy; 8 Department of Physics, University of Calabria, via ponte Bucci, cubo 31/C, 87036 Rende, Italy; lorenzo.caputi@fis.unical.it (L.S.C.); gennaro.chiarello@fis.unical.it (G.C.)

**Keywords:** indium selenide, exfoliation, Bridgman-Stockbarger growth, chemical reactivity, angle-resolved photoemission spectroscopy, nanodevices

## Abstract

Among the various two-dimensional semiconductors, indium selenide has recently triggered the interest of scientific community, due to its band gap matching the visible region of the electromagnetic spectrum, with subsequent potential applications in optoelectronics and especially in photodetection. In this feature article, we discuss the main issues in the synthesis, the ambient stability and the application capabilities of this novel class of two-dimensional semiconductors, by evidencing open challenges and pitfalls. In particular, we evidence how the growth of single crystals with reduced amount of Se vacancies is crucial in the road map for the exploitation of indium selenide in technology through ambient-stable nanodevices with outstanding values of both mobility of charge carriers and ON/OFF ratio. The surface chemical reactivity of the InSe surface, as well as applications in the fields of broadband photodetection, flexible electronics and solar energy conversion are also discussed.

## 1. Introduction

The recent interest toward layered semiconductors [1,2,3,4,5] is motivated by their potential impact in nanoelectronics [6,7,8,9], due to the joint presence of finite values of band gaps [10,11] and flexibility [12,13]. In particular, while graphene does not have a band gap [14,15,16,17], van der Waals semiconductors enable the devising of nanodevices with outstanding values for the ON/OFF ratio [6]. Moreover, by reducing the thickness, in some cases, the band gap becomes direct, with implications for optoelectronics [18] and photodetection.

Indium selenide represents an intriguing candidate for nanoelectronics [19], since it is an ambient-stable [20], flexible [21,22,23] semiconductor with high values of mobility of charge carriers [24]. Moreover, by exfoliating the bulk crystal, it is possible to attain nanosheets with a highly crystalline quality [25,26,27]. 

InSe is made of stacked layers of Se-In-In-Se atoms with van der Waals bonding between quadruple layers [28,29]. Recently, several researchers have reported the outstanding efficiency of InSe-based optoelectronic devices [30,31,32]. Field-effect transistors (FETs) with an active channel of few layers of InSe are characterized by values of electron mobility at room temperature as high as 10^3^ cm^2^/(V·s) [30]. Furthermore, InSe has good prospects for applications in the field of photovoltaics [22], strain engineering [33], and nonlinear optics [34].

**Figure 1:**
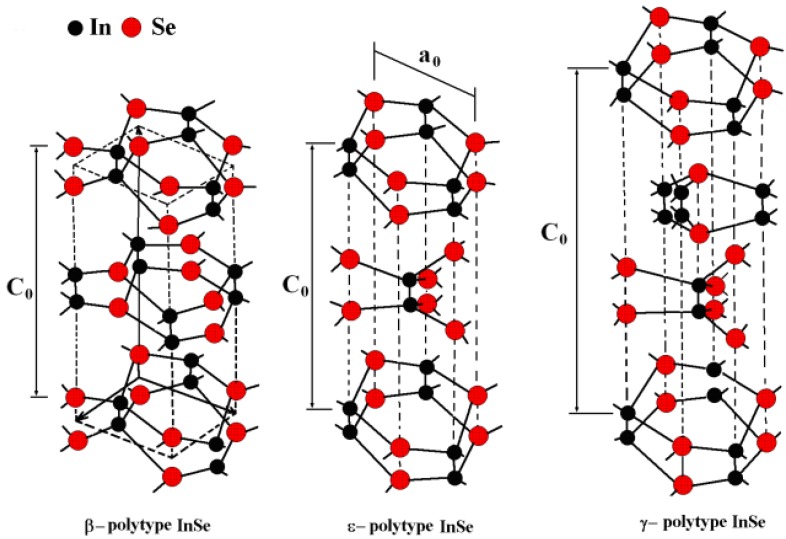
Geometrical structure of the three polytypes of InSe. C_0_ and a_0_ represent the lattice parameters along the perpendicular to layers and in the layer plane, respectively. Reproduced from Ref. [39].

Depending on the different stacking sequences of layers, different polytypes of layered materials exist. Three highly distinct polytypes of the InSe crystal have been identified [35,36] (β, ε, and γ, see Figure 1), in which In and Se atoms are differently arranged. The β (space group symmetry $D_{6h}^{4}$) and ε polytypes (space group symmetry $D_{3h}^{1}$) are characterized by a hexagonal lattice consisting of eight atoms in the unit cell, and their extension over two layers [37]. Rhombohedral γ polytype (space group symmetry $C_{3v}^{5}$) contains two cations and two anions distributed on four adjacent layers [35,38].

While ε-InSe has an indirect band gap of 1.4 eV [36], both β- and γ-InSe have a direct band gap [36] with closely matching values (1.28 eV [37] and 1.29 eV [40], respectively). Accordingly, only β and γ phases of InSe could supposedly be employed in optoelectronics, for which finite and direct band gaps are beneficial [41]. Exfoliated samples of γ-InSe host quantization effects, which enable near-infrared photoluminescence emission [26].

## 2. Growth

Several growth techniques have been used for growing indium-selenide compounds: vacuum evaporation [42], molecular beam epitaxy (MBE) [43], colloidal methods [44,45], flash evaporation [46], chemical vapour deposition [47], van der Waals epitaxy [48], radiofrequency sputtering [49], Czochralski [50] and the Bridgman-Stockbarger technique [37,51,52]. 

**Figure 2:**
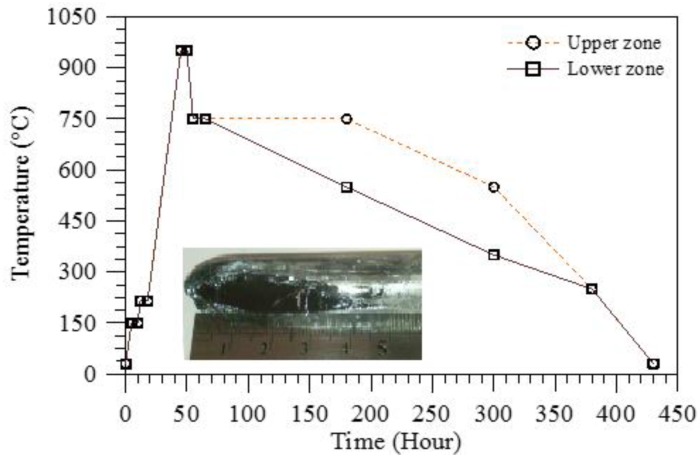
Temperature profile of the furnace in a typical Bridgman-Stockbarger growth of InSe single crystals [37]. In the inset an InSe single-crystal ingot grown by the Bridgman/Stockbarger method is displayed.

Among these methods, the Bridgman-Stockbarger growth is the most suitable for the large-scale production of high-quality bulk InSe semiconductors for device applications, with a typical time requirement ranging from 5 to 35 days for an ingot. The quite long time needed is also a consequence of some technical difficulties that need to be overcome during the growth process. The Bridgman-Stockbarger method involves starting from high-purity In and Se elements, which are sealed in quartz ampoules, heated in vacuum in a furnace at temperatures as high as 950 °C. Figure 2 shows the typical temperature profile at which the furnace should be kept during the growth process. However, selenium has a high vapor pressure value at temperatures higher than 600 °C [53], with possible fractures on the bulbs being used for growth. This problem can be avoided by decreasing the reaction rate, increasing the growth time, and/or by decreasing the growth temperature with a subsequent slower growth rate. Successively, the crucible is suspended in the middle of the vertical furnace with two designated zones. The temperature of the lower zone of the furnace is reduced to ~250 °C at a rate typically of around 1.5 °C/h. Both furnace zones are cooled to 250 °C over ~75 h. The solidified ingot is then cooled to room temperature over ~50 h.

Using the Bridgman-Stockbarger method, InSe single crystals can be grown as both n- and p-type depending on growth conditions and dopant elements [54]. Doping can be achieved by the direct addition of the dopant elements to the growth ampoules [55,56,57,58,59,60,61,62,63,64,65,66,67]. To introduce dopants in a homogeneously distributed fashion, during the growth it is necessary to rotate the growth furnace, kept at about 50 °C above the melting point, around its own axis for many hours, at a particularly slow rate. In the choice of the dopant, one has to consider that the atomic radius of the doped elements should match that of the replaced atoms. In general, the grown crystals of InSe inevitably contain numerous defects, which act as deep trap levels. We reiterate that defects and impurities in semiconductors are associated with the energy levels in the forbidden gap. Thus, the presence of Se vacancies, i.e., anion vacancies, in InSe crystals leads to deep energy levels in the band gap. By doping InSe with transition metals, the localized levels originating from the vacancies disappear [37].

## 3. Exfoliation

The ingots of InSe, obtained by Bridgman-Stockberger technique, have no cracks and voids at the surface. Due to the extreme weakness of the interlayer van der Waals bond, the ingots can be easily cleaved along the (001) planes. Flakes with outstanding flatness and bright surfaces can be exfoliated from the parental bulk single-crystal ingot without chemical etching or mechanical polishing processes. To date, only mechanical exfoliation [23,26] (the ordinary scotch tape method) has been used to obtain atomically thick flakes of InSe. It is expected that liquid-phase exfoliation could be also achieved in future works on InSe. This achievement would boost the technological exploitation of InSe. 

## 4. Electronic Properties

**Figure 3:**
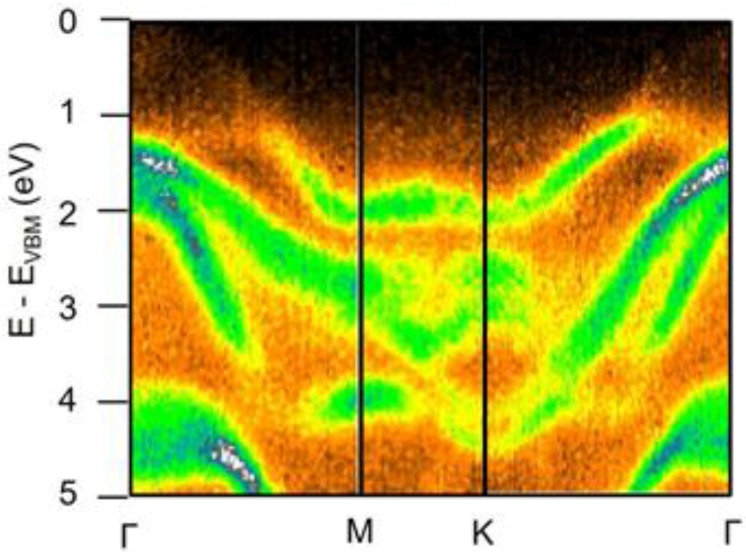
Experimental band structure of β-InSe along the high-symmetry directions. The energy scale was set to zero at valence-band maximum (VBM). Reproduced with permission from Ref. [68].

The electronic band structure of β-InSe has been measured by angle-resolved photoemission spectroscopy (ARPES) only in the case of bulk crystals [68] (Figure 3).

**Figure 4:**
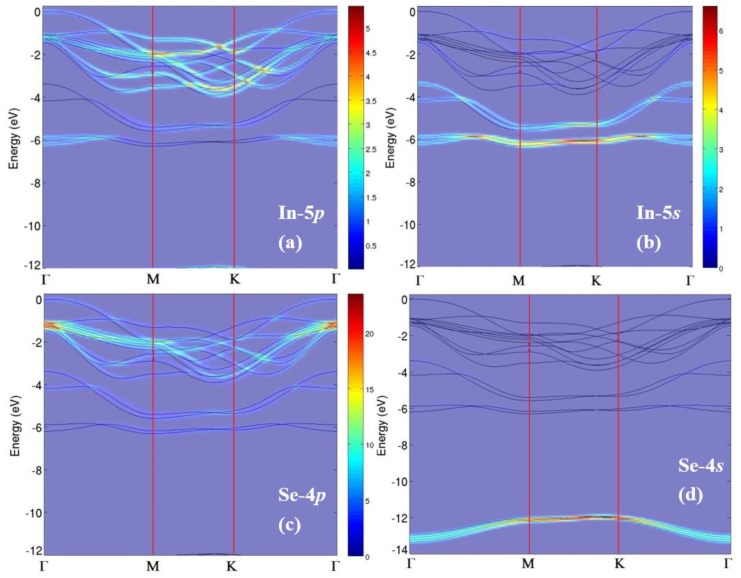
Theoretical band structure projected to (**a**) In-5p; (**b**) In-5s; (**c**) Se-4p; and (**d**) Se-4s atomic orbitals. The intensity of the various bands is reported in a color scale, whose legend is displayed in the right part of panels (**a**,**b**) and between panels (**c**,**d**). Reproduced with permission from Ref. [68].

**Figure 5:**
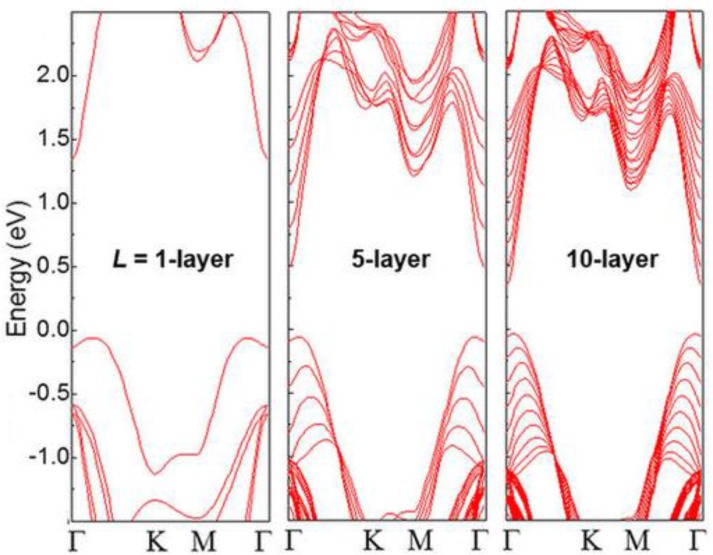
Band structure of γ-InSe with a number of layers *L* = 1, 5 and 10 layers. Reproduced with permission from Ref. [69].

The orbital components of the different bands experimentally revealed by ARPES are indicated by projections to 5p (panel a of Figure 4) and 5s (panel b) states of In and to 4p (panel c) and 4s (panel d) states of Se along the whole Brillouin zone.

The valence-band maximum (VBM) is mainly derived from 5p_z_ states of In. The crossing of bands at ~1.6 eV at Γ principally originates from the Se-4p and In-5s states, whereas states at 4.5 eV predominantly arise from In-5s. 

**Figure 6:**
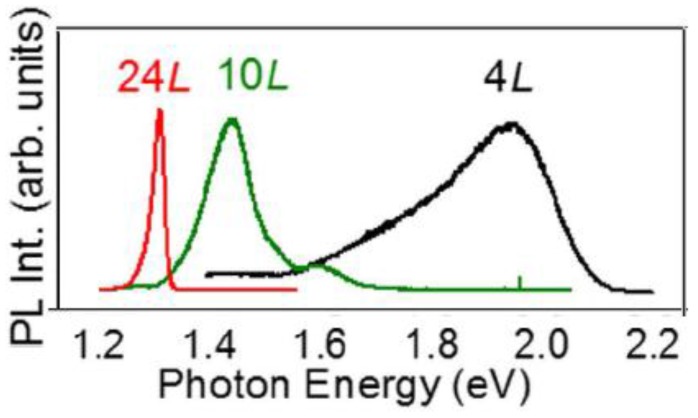
Normalized microphotoluminescence spectra for γ-InSe nanosheets with *L* = 4, 10 and 24 layers. Reproduced with permission from Ref. [69].

Concerning γ-InSe, density functional theory has evidenced a transition from direct to indirect band gap semiconductor, which occurs by reducing the number of layers [69], as also manifest from the analysis of Figure 5, where the behavior of the band structure of γ-InSe as a function of the thickness is shown. Figure 6 displays the dependence of the photoluminescence spectra, acquired with micrometric spatial resolution, as a function of thickness; by reducing the thickness, a shift of the maximum toward higher photon energies occurs.

## 5. Ambient Stability

**Figure 7:**
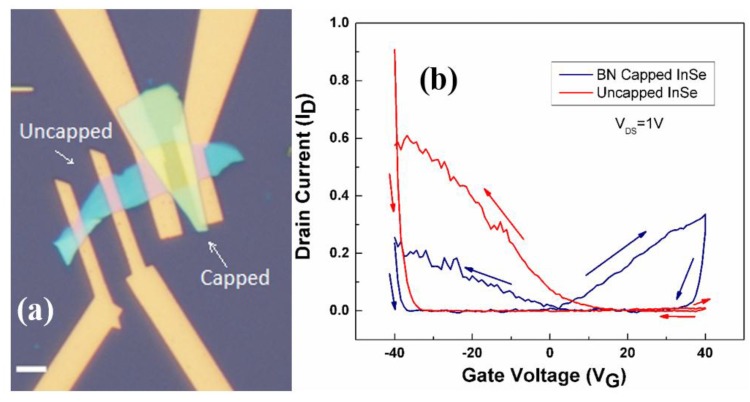
(**a**) Optical image of typical InSe back-gate transistor devices: in one device the channel is capped with a flake of hexagonal boron nitride (h-BN), whereas the other channel is exposed to atmosphere. (**b**) Behavior of the drain current as a function of the gate voltage for the cases of capped and uncapped InSe-based transistors. The uncapped device shows dominant p-type transport (even if with notable hysteresis), while the capped device noticeably displays ambipolar transport. Reproduced with permission from Ref. [20]. Copyright Royal Society of Chemistry, 2016.

The stability of the performances of nanodevices in ambient conditions is an essential requisite in order to devise applications based on InSe. The ambient stability of FETs with an active channel of InSe has been demonstrated in Ref. [20]. However, therein, it was also shown that FETs with an active channel of uncapped InSe exhibit a p-type transport, while ambipolar transport was achieved only in the presence of a capping layer (Figure 7). 

**Figure 8:**
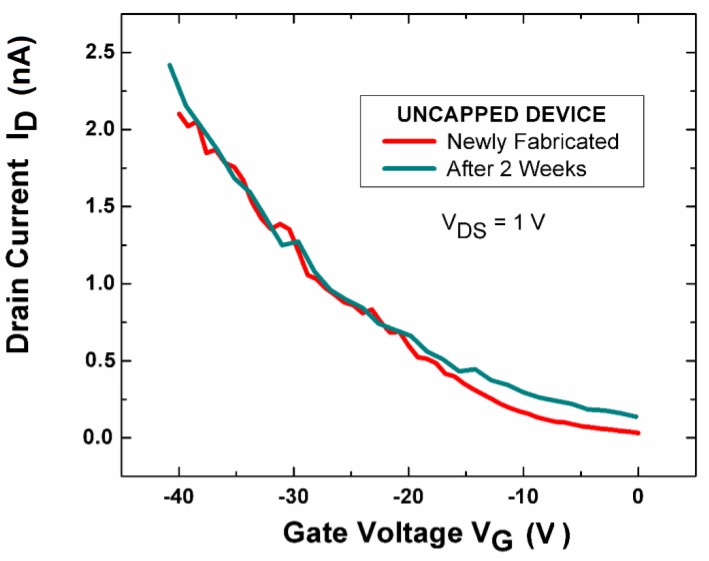
*I*_DS_-*V*_G_ curve of uncapped InSe-based transistors freshly fabricated (red curve) and after two weeks (blue curve). Reproduced with permission from Ref. [20]. Copyright Royal Society of Chemistry, 2016.

Nevertheless, even the uncapped InSe nanodevices are stable in air without any perceptible modification in the *I*-*V* curves measured again after two weeks (Figure 8). Consequently, one can affirm that no fast degradation occurs for InSe, contrary to the cases of Bi_2_Se_3_ [70] and black phosphorus [71]. 

**Figure 9:**
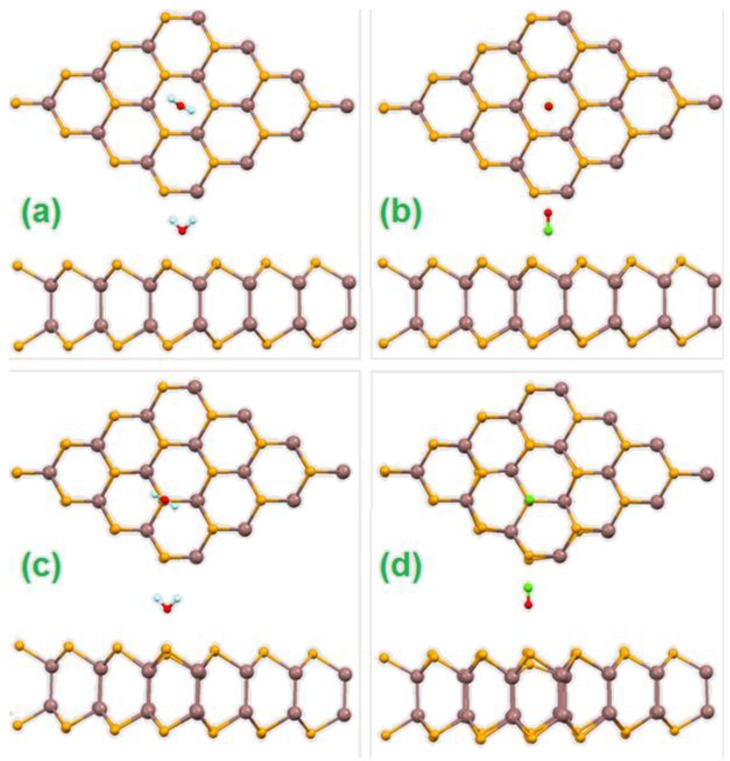
Top and side views of the geometrical structure of InSe monolayer in the case of adsorption of H_2_O (**a**,**c**) and CO (**b**,**d**) molecules over hole sites (**a**,**b**) and top sites (**c**,**d**) with respect to Se atoms. Reproduced with permission from Ref. [20]. Copyright Royal Society of Chemistry, 2016.

For the case of InSe, the origin of the p-type doping of ambient-stable InSe-based FETs was unveiled in Ref. [20], where the authors investigated the correlation between the surface chemical reactivity, the environmental doping and the effects induced by defects in InSe (Figure 9).

The interaction of ambient gases (H_2_O, CO, CO_2_, N_2_, O_2_) with InSe was assessed for two adsorption sites on undefected InSe: over Se atoms, which are the outermost surface atoms of the InSe monolayer (top position, Figure 9a,b), and over the middles of hexagons (hole position, Figure 9c,d). Theoretical results (Table 1) clarify that for all examined molecules the preferential adsorption site is that over holes. 

**Figure 10:**
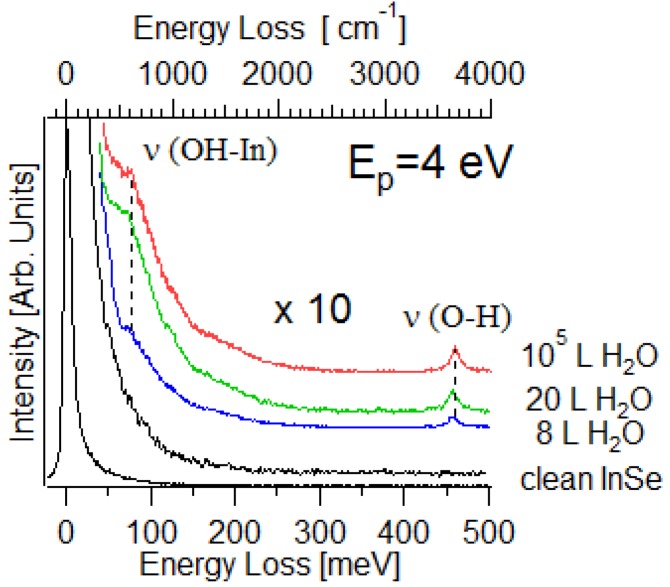
Vibrational spectrum of InSe exposed to water molecules at room temperature. Reproduced with permission from Ref. [20]. Copyright Royal Society of Chemistry, 2016.

According to the values of adsorption energies calculated in Table 1, only the adsorption of H_2_O on hole sites is energetically favorable at room temperature, with a charge transfer of 0.01 electrons per each molecule. As a result, the InSe surface is only slightly p-doped. Therefore, among ambient gases, only water is able to form stable bonds with InSe at room temperature with manifest displacement of Se atoms from their positions in the pristine InSe (Figure 9c,d). These theoretical predictions have been confirmed by vibrational experiments, which also indicated that the adsorption of water molecules on InSe at room temperature is entirely dissociative. The vibrational spectrum of water-dosed InSe (Figure 10) exhibits an intense band at 450 meV, which constitutes a fingerprint of the water decomposition that occurred. As a matter of fact, this mode is due to the O–H stretching vibration in –OH groups [72], while molecular water would display O–H stretching energy in the 400–425 meV range [73]. 

Similarly, other recent theoretical works have recently reported positive adsorption energy of water molecules on Se vacancies [74] and almost zero adsorption energies for the physical adsorption of oxygen over various adsorption sites of the InSe surface [75]. Other theoretical works [76,77] Have reported rather large (0.1~0.3 eV) negative values of adsorption energies, but disagree with each other about type of doping from NH_3_ and about the values of the transferred charge from InSe substrate to molecules. In particular, results in Ref. [77] point to stable adsorption of molecules from air for all adsorption sites of InSe and, according to these theoretical predictions, InSe should be much more heavily doped under ambient conditions. However, it is quite evident that the theoretical model by Ma et al. [77] disagrees with experiments.

In InSe, extreme sensitivity of adsorption properties to local distortions is expected. Figure 9 depicts how adsorption on different sites provides different distortions of the atomic structure of InSe substrate. Molecular dynamic simulations also demonstrate the influence of the water layer on the structure of the InSe monolayer [78]. The optimization of both atomic positions and lattice constants provides a different magnitude of local distortions and, as a result, charge distribution, adsorption energies and charge transfer are subsequently modified. 

It is worth reporting that two computational studies (both using the Vienna Ab initio simulation package- VASP- code) have recently reported contradictory results on the change of the lattice parameter when the InSe thickness is reduced down to the monolayer regime [79,80]. This disagreement between theoretical models highlights the noticeable dependence of results of the calculations on various usually unimportant and technical parameters. Moreover, the dependence of the energetics of adsorption from distortions permits the manipulation of chemical and electric properties of monolayer InSe by strain [32,77] or by distortion of scaffold, as previously reported for graphene [78,79]. Thickness-dependent changes of vibrational spectra of InSe [36] and GaSe [81] also prove the sensitivity of lattice properties of these compounds on the number of layers. Further theoretical investigation of the role of various substrates, ripples and strain on doping of InSe surface is ongoing.

**Figure 11:**
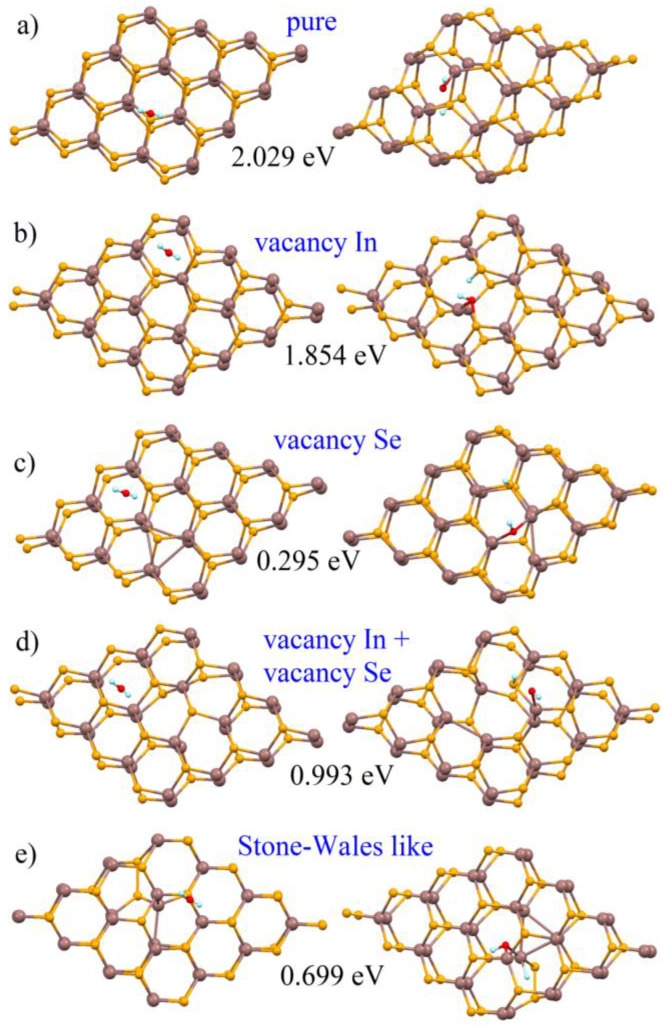
Atomic structure of pristine (**a**) and defective (**b**–**e**) InSe monolayer before (**left**) and after (**right**) the decomposition of the water molecules. The indicated values represent the energy cost of the dissociation of water molecules for each specific case. Reproduced with permission from Ref. [20]. Copyright Royal Society of Chemistry, 2016.

The calculation of the energy cost of dissociation of water molecules in both defect-free and defective InSe monolayer for the various kinds of defects (single In and Se vacancies, the combined existence of single In and Se vacancies, and Stone-Wales defects) indicates that water dissociation over undefected InSe is not energetically favored. In detail, the energetic cost is ~2 eV (Figure 11a). Analogously to the case of graphene [82], the presence of defects noticeably reduces the energy cost of chemisorption by a value depending on the type of defect. The presence of In vacancies reduces the energy cost for water decomposition to 1.85 eV (Figure 11b). Water decomposition is particularly favorable on Se vacancies, over which the energy cost is only 0.29 eV (Figure 11c). For the case of the joint presence of single In and Se vacancies, the energy cost for decomposition is about 1 eV (Figure 11d), while it is ~0.7 eV for the case of Stone-Wales defects (Figure 11e).

Based on the relationship between calculated DFT energies and temperatures of reactions [83], it can be concluded that reactions with energies below 0.5 eV, e.g., in the case of water decomposition at Se vacancies, occur at room temperature at a rather high rate [20]. Calculations by Shi et al. [74] also indicate favorability of water decomposition on Se vacancies, and suggest a possible solution for this problem by reparation of the vacancies through substitution of missing Se atoms by S with a treatment of InSe surface in CH_3_SH. It is worth noticing that the substitution of Se with S and also the formation of local In_2_O_3_-like structures resulting from the penetration of oxygen atoms into the InSe monolayer [75] do not provide any visible changes in the band structure of InSe. These results suggest the routes toward healing of defects in InSe surfaces, but further modeling is required to find realistic methods.

## 6. Applications

**Figure 12:**
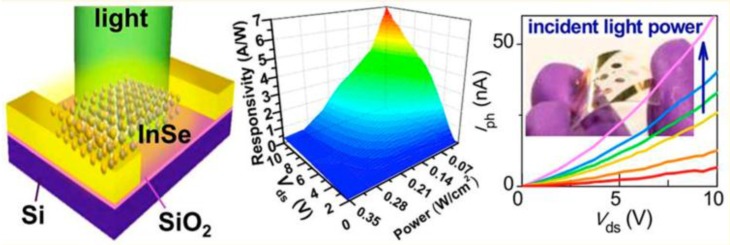
(**left panel**) Sketch of a InSe-based FETfor optoelectronics; (**middle panel**) Behavior of the responsivity as a function of the power and of the drain-source voltage; (**right panel**): The photocurrent of the InSe device on PET film (shown in the inset in the bent geometry) acquired when the device was in planar geometry with 633 nm illumination of 22.74, 8.67, 4.46, 2.85, 0.70, and 0.29 mW·cm^−2^ for purple, blue, green, yellow, orange, and red curves, respectively. Reproduced with permission from Ref. [23]. Copyright American Chemical Society, 2014.

Compared with other group IIIA-VIA layered (*MX*, *M* = Ga and In, *X* = S, Se and Te), InSe has a narrower bandgap, which overlaps well with the solar spectrum and offers efficient solar energy conversion. Several works [84,85,86,87,88] have already demonstrated that InSe is an alternative candidate to thin-film cells, due to its high mechanical flexibility [21,23]. Tamalampudi et al. [23] have demonstrated that photodetectors with an active channel of a few layers of InSe show broadband efficiency from the visible to the near-infrared range (450–785 nm). InSe-based photodetectors have been fabricated on (i) a rigid SiO_2_/Si substrate, and (ii) a flexible polyethylene terephthalate (PET) film [23]. Photoresponsivities as high as 12.3 A·W^−1^ at 450 nm (on SiO_2_/Si) and 3.9 A·W^−1^ at 633 nm (on PET) have been measured [23], and with an order of magnitude improvement by simply sweeping the gate voltage above threshold of the transfer characteristic for electron conduction. The responsivity of InSe-based photodetectors in Figure 12 goes up to 7 A·W^−1^ at power intensity lower than 0.07 W·cm^−2^ (λ~633 nm), and this value is substantially higher than that of graphene- (5 × 10^−4^ A·W^−1^) and MoS_2_- (4.2 × 10^−4^ A·W^−1^) based photodetectors. Thus, InSe-based photodetectors are the most efficient among those realized with two-dimensional materials, including graphene and transition-metal dichalcogenides [7]. Their response time is only a few tens of ms and, moreover, they show long-term stability in photoswitching [23]. Remarkably, the bending of the InSe-based device fabricated on the flexible PET substrate does not significantly reduce its performances [23]. Therefore, the stretchable nature of InSe represents an ideal candidate for advanced optoelectronic applications.

With such excellent optoelectronic merits, layered InSe nanosheets will become not only outstanding candidates for optical sensing applications, but also promising components for configuring 2D heterostructure devices for high-performance photodetectors and emitters [89]. Owing to the low-density interface states, InSe-based hetero-junctions can be used for tailoring the device characteristics [90]. A graphene/few-layer InSe heterostructure photodetector has been reported with an external quantum efficiency of over 2 × 10^5^%, which represents a spectacular improvement with respect to the homostructural few-layer InSe device [32]. An absolute power conversion efficiency of about 18% for wavelengths of 1.10–1.25 µm has been reported for solar cells exploiting crystalline InSe as a window layer in heterostructure diodes [91]. Homojunction diodes formed from layers of p- and n-type InSe exhibit electroluminescence of exciton recombination at *hν*~1.23 eV. A ~20 meV redshift with respect to the photoluminance peak (1.25 eV) is observed. This redshift is attributed to the reabsorption of photons by the top InSe layer [92]. Conversely, heterojunction diodes formed by combining layers of p-type GaSe and n-type InSe emit photons at lower energies, due to the generation of spatially indirect excitons and a staggered valence band lineup for the holes at the GaSe/InSe interface [93].

Finally, in the prospect of a technological employment of atomically thin InSe nanosheets, it is worth mentioning that, by means of morphological nano-manipulation procedures such as nanotexturing, it is possible to enhance light absorption, bandwidth and the luminescent response [94].

## 7. Conclusions

In this feature article, we have discussed the main features of the science and technology based on InSe. Single crystals of InSe can be grown by different techniques, among which Bridgman-Stockbarger is the most suitable for up-scaling. As a matter of fact, it allows the production of large-scale, high-quality single crystals, which can be easily exfoliated to atomic thickness. To date, only mechanical exfoliation has been used, but we suggest that liquid-phase exfoliation would allow deeper employment of InSe in technology.

Nanodevices with flakes of InSe as active channels are characterized by high mobility of charge carriers and very high ON/OFF ratios. By reducing the thickness of the flakes, the band gap energy increases and a direct-to-indirect transition occurs, while electron and exciton effective masses are substantially constant with thickness. Concerning potential applications of InSe, highly performing broadband photodetectors and solar cells have been realized. The flexibility of InSe makes it a solid candidate for flexible electronics.

Ambient stability is one of the most valuable properties of InSe. However, the presence of Se vacancies enables water decomposition at room temperature, with the introduction of a p-type doping in InSe exposed to ambient air humidity. Therefore, the minimization of defects is the most important challenge in the growth of InSe compounds.

## Figures and Tables

**Table 1:** Adsorption energies of different ambient species over the hole and on-top with respect to Se atoms. Reproduced with permission from Ref. [20]. Copyright Royal Society of Chemistry, 2016.

Species/Adsorption Site	Hole (eV)	Top (eV)
H_2_O	−0.139	0.69
CO	0.014	2.75
CO_2_	0.244	2.05
N_2_	0.097	1.15
O_2_	0.705	3.67
